# Transactions of the College of Physicians, of Philadelphia

**Published:** 1875-10

**Authors:** 


*Transactions of the College of Physicians, of Philadelphia,* Third Series, Vol. 1. Printed for the College, 1875.

Among the many interesting and instructive reports published in this volume, is a complete history of the Autopsy on the Bodies of Chang and Eng Bunker, commonly known as the "Siamese Twins," illustrated by chromo lithographs, and wood cuts.

